# Sensitive and Specific Detection of *Trypanosoma cruzi* DNA in Clinical Specimens Using a Multi-Target Real-Time PCR Approach

**DOI:** 10.1371/journal.pntd.0001689

**Published:** 2012-07-03

**Authors:** Yvonne Qvarnstrom, Alejandro G. Schijman, Vincent Veron, Christine Aznar, Francis Steurer, Alexandre J. da Silva

**Affiliations:** 1 Division of Parasitic Diseases and Malaria, Center for Global Health, Centers for Disease Control and Prevention, Atlanta, Georgia, United States of America; 2 LabMeCh, INGEBI-CONICET, Buenos Aires, Argentina; 3 Laboratoire hospitalier et Universitaire-CH Andrée Rosemon, Faculté de Médecine H. Bastaraud-EA 3593, Cayenne, Guyane Française; University of Pittsburgh, United States of America

## Abstract

**Background:**

The laboratory diagnosis of Chagas disease is challenging because the usefulness of different diagnostic tests will depend on the stage of the disease. Serology is the preferred method for patients in the chronic phase, whereas PCR can be successfully used to diagnose acute and congenital cases. Here we present data using a combination of three TaqMan PCR assays to detect *T. cruzi* DNA in clinical specimens.

**Methods/Principal Findings:**

Included in the analysis were DNA extracted from 320 EDTA blood specimens, 18 heart tissue specimens, 6 umbilical cord blood specimens, 2 skin tissue specimens and 3 CSF specimens. For the blood specimens both whole blood and buffy coat fraction were analyzed. The specimens were from patients living in the USA, with suspected exposure to *T. cruzi* through organ transplantation, contact with triatomine bugs or laboratory accidents, and from immunosuppressed patients with suspected Chagas disease reactivation. Real-time PCR was successfully used to diagnose acute and Chagas disease reactivation in 20 patients, including one case of organ-transmitted infection and one congenital case. Analysis of buffy coat fractions of EDTA blood led to faster diagnosis in six of these patients compared to whole blood analysis. The three real-time PCR assays produced identical results for 94% of the specimens. The major reason for discrepant results was variable sensitivity among the assays, but two of the real-time PCR assays also produced four false positive results.

**Conclusions/Significance:**

These data strongly indicate that at least two PCR assays with different performances should be combined to increase the accuracy. This evaluation also highlights the benefit of extracting DNA from the blood specimen's buffy coat to increase the sensitivity of PCR analysis.

## Author Summary

Chagas disease is endemic in several Latin American countries and affects approximately 8 to 11 million people. The protozoan parasite, *Trypanosoma cruzi*, is the agent of Chagas disease, a zoonotic disease that can be transmitted to humans by blood-sucking triatomine bugs. Other routes of infection include congenital transmission, blood transfusion, organ transplantation, accidental inoculation of the parasite during laboratory research and by consuming food and juice contaminated with the parasite. This study focused on the evaluation of three quantitative PCR (QPCR) assays for the diagnosis of Chagas disease. The evaluation was based on the analysis of 349 specimens submitted for confirmatory diagnosis of Chagas disease to the Centers for Disease Control and Prevention from 2008 to 2010. By using such assays we were able to diagnose acute and Chagas disease reactivation in 20 patients, including one case of organ-transmitted infection and one congenital case. The paper also highlights the benefit of extracting DNA from the blood specimen's buffy coat to increase the sensitivity of diagnostic PCR analysis. The results obtained in this study strongly indicate that at least two QPCR assays with different performance characteristics should be combined to increase diagnostic accuracy.

## Introduction

Chagas disease is a vector-borne infectious disease caused by the parasite *Trypanosoma cruzi*. It is endemic in several countries of Central and South America. In endemic areas the disease is spread by certain species of triatomine bugs that excrete the parasites in their feces while feeding on human hosts. Humans get infected when feces from infected triatomines contaminates wounds, allowing the parasite to enter the bloodstream. Other routes of infection include congenital transmission, blood transfusion, organ transplantation, accidental inoculation of the parasite during laboratory research and by consuming food and juice contaminated with the parasite. As efforts to control vector-borne and blood transmission are successful, congenital and oral transmission paths are becoming increasingly important [1].

After a short acute phase when the parasite can be found circulating in the blood, the disease enters the chronic phase when the amastigote stage develops and multiplies in organ tissues, primarily in the heart. The chronic phase is characterized by two forms; patients first develop the indeterminate form of chronic infection which can last for decades and the patients are typically asymptomatic during this time. An estimated 30–40% of patients may develop clinical disease, with manifestations such as cardiomyopathy or digestive megasyndromes [1]. Chronically infected patients that become immunosuppressed may experience a reactivation of the disease, a condition characterized by increasing parasitemia and atypical presentations such as epidermic lesions and compromise of the central nervous system [2].

The options for laboratory diagnosis of Chagas disease depend on the disease phase. Serology is the method of choice to diagnose chronic infections. Acute infections can be diagnosed by detecting motile organisms in fresh blood preparations, by culture or by detection of parasite DNA by PCR [3]. The latter methods are also recommended to detect increasing parasitemia in cases of reactivation following immunosuppression [4]. In cases of acute infections or reactivation of chronic disease it is important to use sensitive diagnostic methods since early detection and treatment results in a more favorable outcome. PCR-based methods are generally considered to be more sensitive than microscopy and have lately been increasingly used to diagnose Chagas disease [4]. However, the use of PCR is also challenging as there is no “gold standard” method for the diagnosis of Chagas disease [5], [6] and the diagnostic performance can vary widely depending on the type of PCR assay. The most widely used PCR assays used for diagnostic purposes target either the kinetoplast genome (kDNA), also called the minicircle, or a nuclear mini-satellite region designated TCZ [4], [6], [7], [8], [9], [10], [11], [12], [13], [14], [15], [16]. Both of these targets are present in multiple copies in the parasite genome, which increases the sensitivity of detection [15], [17]. However, assays that target these regions have been reported to cross-amplify non-*T. cruzi* DNA [10], [16], [18], [19], [20]. Assays that amplify other genes may show better specificity but they are generally less sensitive [4], [9], [16], [21].

One important use of PCR as a diagnostic tool is to provide a sensitive method to detect reactivation in chronically infected patients with immunosuppression. Patients with chronic Chagas heart disease often require a heart transplant [22]. Current recommendations state that these patients should be monitored at regular intervals after the transplant for signs of increasing parasitemia [3]. Another category of patients for whom PCR testing is beneficial is patients who receive organs from chronically infected donors. Since only a fraction of organ recipients will develop an acute *T. cruzi* infection, preventive drug treatment is not recommended. In such cases the use of PCR can allow for early detection of those cases where transmission has occurred.

Recently, an international collaborative study focusing on standardization and validation of PCR for diagnostic detection of *T. cruzi* DNA was conducted [13]. The study relied on the use of DNA specimens from genetically distinct cultured *T. cruzi* strains plus blood specimens from chronically infected patients. The specimens were coded at a coordinating laboratory and shipped to 26 participating laboratories that performed PCR testing according to their own standard operating procedures. Results were then sent back to the coordinating laboratory and performance characteristics were calculated for each PCR assay. The study found a high degree of variability in accuracy and performance among the included PCR tests and identified and further evaluated two DNA extraction methods and four PCR assays that performed better than the others. Two of the best-performing assays were real-time PCR assays.

To continue these efforts we here present results from a diagnostic testing algorithm involving three of the real-time PCR assays included in the international validation study mentioned above. Real-time PCR has several advantages over conventional PCR, e.g. shorter turnaround times and less risk of amplicon carry-over contamination [23], both of which can be advantageous in diagnostic laboratories. One of the real-time PCR assays included in this study was ranked among the four best-performing assays in the international validation study; a real-time PCR assay targeting the mini-satellite TCZ region. The second real-time PCR assay was selected because it was the best-performing real-time PCR assay targeting the kDNA included in the international validation study. The third real-time PCR assay was included in this study because it targets the small subunit ribosomal RNA (18 S rRNA) gene, which is generally suitable for diagnostic assays because it is highly conserved.

In contrast to the international validation study we mainly used specimens from patients with suspected acute or reactivating Chagas disease since PCR testing is more relevant for early diagnosis or monitoring in this patient group than in chronic patients, whose diagnosis relies on serological methods. The majority of the specimens tested were EDTA blood samples; we performed real-time PCR on DNA extracted from buffy coat preparations in addition to whole blood to determine the effect of buffy coat concentration on the sensitivity of the PCR analysis.

## Methods

### Clinical specimens

All the specimens used in this study were submitted to CDC for confirmatory diagnosis of Chagas disease during years 2008–2010 from state public health laboratories, hospitals and private clinics in the United States. The tests were performed on 349 laboratory specimens from 119 patients, who lived in the United States at the time of specimen collection. A breakdown of the specimen types and the conditions that prompted the diagnostic requests are presented in Table 1. Samples analyzed in this study were anonymized by removing identifiers after diagnostic results were reported, in accordance with the CDC IRB, protocol number 3580, entitled “Use of Human Specimens for Laboratory Methods Research”. All of the patients included in this study were evaluated for serology status using the Chagatest recombinante v. 3.0 (Wiener Laboratorios, Rosario, Argentina) and a CDC in-house IIF test.

**Table 1:** Specimens included in this study.

reason for testing	number of patients	Number of specimens					
		EDTA blood	cord blood	heart tissue	skin tissue	CSF	total
reactivation due to heart transplant	18	63	0	15	1	0	**79**
reactivation due to AIDS	5	6	0	0	0	2	**8**
reactivation due to other condition	2	5	0	0	0	0	**5**
symptoms of cardiomyopathy	13	10	0	3	0	0	**13**
symptoms of acute Chagas disease	18	26	0	0	1	1	**28**
bites from triatomines but symptom-free	13	13	0	0	0	0	**13**
congenital transmission	20	16	6	0	0	0	**22**
recipients of organs from Chagas positive donors	14	139	0	0	0	0	**139**
laboratory accidents	9	35	0	0	0	0	**35**
serologically positive but symptom-free*	7	7	0	0	0	0	**7**
**total**	**119**	**320**	**6**	**18**	**2**	**3**	**349**

*:  = these included three mothers in investigations of possible congenital transmission and four organ donors with chronic Chagas disease. PCR-testing was performed on these chronically infected persons to aid in the evaluation of disease transmission risk.

### DNA extraction

DNA extraction was performed from all specimens within 24 hours of arrival at the laboratory. DNA was extracted from whole blood specimens using the QIAamp blood mini DNA kit (QIAGEN, Valencia, Calif.). The volume of whole blood used was 0.2 ml and if the remaining volume exceeded 1 ml, the buffy coat fraction was separated as follows: up to 2 ml of whole blood was centrifuged at 2,500×g for 10 minutes. The plasma was removed and the buffy coat layer plus some of the erythrocyte pellet were transferred to a clean tube. DNA was then extracted from that material in parallel with the whole blood aliquot using the same method mentioned above. Three EDTA blood specimens had enough volume left after initial DNA extraction to allow for one or more additional buffy coat preparations. Two-ml aliquots of these specimens were stored at 4°C for one, two or four weeks and then processed as described above. DNA from tissue specimens was extracted with the DNeasy blood and tissue DNA kit (QIAGEN). For cerebrospinal fluids (CSF), approximately half of the total volume received (0.5–1 ml) was centrifuged for 5 minutes at 6000×g. Most of the supernatant was carefully removed until 0.2 ml remained and DNA was extracted from this remaining volume (plus any pellet) with the DNeasy blood and tissue DNA kit (QIAGEN). All the DNA extraction procedures were performed following manufacturer's instructions for the different types of samples. Previous experiences with these methods in our laboratory had ensured that they efficiently removed potential PCR inhibitors from the specimen types included in this study (data not shown). One negative extraction control was included in each batch of DNA extractions to monitor for potential cross- contamination among samples and contamination of kit reagents.

### PCR protocols

All three real-time PCR assays were included in the international validation study [13]. Table 2 summarizes validation data for the PCR assays as presented in that study, plus specificity data for two other *Trypanosoma* spp. obtained in our laboratory. The real-time PCR assays were performed and analyzed in an Mx3000P QPCR system (Agilent Technologies, Calif.). Each DNA sample was added to the PCR mix in two different concentrations (corresponding to 5 µl and 1 µl of undiluted DNA). All PCR runs included two or more negative amplification controls (adding water instead of template DNA) plus two positive amplification controls (DNA extracted from a culture of the Y strain in two different dilutions). The risk of false positive results due to contamination was minimized by the following procedures: using separate rooms for DNA extraction, pre-and post-amplification processes; having a uni-directional workflow; and using enzymatic removal of contaminating amplicons before real-time PCR amplification.

**Table 2:** Validation data for the real-time PCR assays.

	kDNA TaqMan	TCZ TaqMan	18 S rRNA TaqMan	reference
diagnostic sensitivity (using serology as comparison method)	78%	63%	6%	[13]
diagnostic specificity (using serology as comparison method)	40%	100%	100%	[13]
analytical sensitivity (detection limit) for DTU I	0.1 fg/µl	0.1 fg/µl	10 fg/µl	[13]
analytical sensitivity (detection limit) for DTU IV	1 fg/µl	1 fg/µl	10 fg/µl	[13]
DNA from *T. rangeli* cultures (n = 2)	positive	negative	negative	this study
DNA from *T. theileri*-infected tissue (n = 3)	negative	negative	negative	this study

TCZ TaqMan real-time PCR (designated as method LbF1 in the international validation study [13]): This TaqMan assay was performed as described in Piron 2007 [11], except that the Platinum qPCR supermix was used instead of the Universal mastermix from Applied Biosystems.

kDNA TaqMan real-time PCR (designated as method LbG/3 in the international validation study [13]): The reaction mix consisted of 1× Platinum qPCR supermix, 0.4 µM of each PCR primer 32F, 5′-TTT GGG AGG GGC GTT CA-3′, and 148R, 5′-ATA TTA CAC CAA CCC CAA TCG AA-3′, plus 0.1 µM of the LNA TaqMan probe 71P, 5′-CA TCTC AC CCG TACA TT-3′, where the LNA nucleotides [24] are underlined. Total reaction volume was 20 µl. Thermocycling structure was as follows: 2 minute incubation at 50°C to activate UDG degradation, 2 minute incubation at 95°C to activate the hot-start DNA polymerase, and 40 cycles of 95°C for 15 seconds and 58°C for 60 seconds.

18 S rRNA TaqMan real-time PCR (designated as method LbS/4 in the international validation study [13]): The reaction mix consisted of 1× Platinum qPCR supermix, 0.2 µM of each PCR primer TcF1042, 3′-GCA CTC GTC GCC TTT GTG-3′, and TcR1144, 5′-AGT TGA GGG AAG GCA TGA CA-3′ plus 0.05 µM of the TaqMan probe TCP1104, 5′-AA GAC CGA AGT CTG CCA ACA ACA C-3′. Total reaction volume was 20 µl. Thermocycling structure was as follows: 2 minute incubation at 50°C to activate UDG degradation, 2 minute incubation at 95°C to activate the hot-start DNA polymerase, and 40 cycles of 95°C for 15 seconds and 60°C for 60 seconds.

## Results

This study focused on diagnostic specimens tested from 2008 to 2010. Fifty of the 349 samples produced positive results in at least one of the real-time PCR assays. The three real-time PCR assays produced identical results for 329 samples (94%), of which 30 were PCR positive, while the remaining 20 samples had discrepant results in the three assays.

Of the 50 samples with at least one positive PCR result, 37 samples were collected from 13 chronically infected patients with reactivation disease, six samples from three transplant recipients of organs from chronically infected donors, four samples from a patient with acute Chagas disease acquired during travel in an endemic region, one sample from a congenitally transmitted infection and two samples from two patients under evaluation for severe cardiomyopathy. Tables 3, 4, and 5 list the detailed real-time PCR results from specimens collected from a selection of these patients, as outlined below.

**Table 3:** Detailed PCR findings from patients with reactivation disease.

Patient ID	patient description	time of specimen	specimen type	results individual assays			Reported results
				kDNA TaqMan	TCZ TaqMan	18 S rRNA TaqMan	
1	chronic patient with heart transplant	time of heart transplant	explanted heart tissue	**positive**	**positive**	**positive**	positive
		time of heart transplant	heart biopsy	negative	negative	negative	negative
		1 month post-transplant	heart biopsy	**positive**	**positive**	**positive**	positive†
2	chronic patient with heart transplant	2.5 months post-transplant	EDTA blood	**positive**	**positive**	negative	positive
		3 months post-transplant	EDTA blood	**positive**	**positive**	negative	positive†
3	chronic patient with heart transplant	time of heart transplant	EDTA blood	negative	negative	negative	negative
		3 weeks post-transplant	EDTA blood	negative	negative	negative	negative
		5 weeks post-transplant	EDTA blood	**BC positive**	**BC positive**	negative	positive
		6 weeks post-transplant	EDTA blood	**BC positive**	negative	negative	equivocal
		7 weeks post-transplant	EDTA blood	**positive**	**positive**	**positive**	positive
		9 weeks post-transplant	EDTA blood	**positive**	**positive**	**positive**	positive
		3 months post-transplant	EDTA blood	negative	negative	negative	negative†
		7 months post-transplant	EDTA blood	negative	negative	negative	negative
4	chronic patient with heart transplant	time of heart transplant	EDTA blood	**positive**	**positive**	**positive**	positive
		3 months post-transplant	EDTA blood	negative	negative	negative	negative
5	chronic patient with heart transplant	1 week post-transplant	EDTA blood	**positive**	**positive**	negative	positive
		2.5 weeks post-transplant	EDTA blood	**positive**	**positive**	negative	positive
		3.5 weeks post-transplant	EDTA blood	**positive**	**positive**	**positive**	positive
		1 month post-transplant	EDTA blood	**positive**	**positive**	**positive**	positive
		1.5 months post-transplant	EDTA blood	negative	negative	negative	negative†
		5 months post-transplant	EDTA blood	negative	negative	negative	negative
6	chronic patient with heart transplant	time of heart transplant	explanted heart tissue	**positive**	**positive**	negative	positive
		1 week post-transplant	EDTA blood	negative	negative	negative	negative
		2 weeks post-transplant	heart biopsy	negative	negative	negative	negative
		3 weeks post-transplant	heart biopsy	negative	**BC positive**	negative	equivocal
		4 weeks post-transplant	EDTA blood	**BC positive**	**BC positive**	negative	positive
		5 weeks post-transplant	EDTA blood	**positive**	**positive**	**positive**	positive
		3 months post-transplant	EDTA blood	negative	negative	negative	negative†
		6 months post-transplant	EDTA blood	negative	negative	negative	negative
7	chronic patient with organ transplant	1 week post-transplant	EDTA blood	negative	negative	negative	negative
		2 weeks post-transplant	EDTA blood	**positive**	negative	negative	equivocal
		3 weeks post-transplant	EDTA blood	**positive**	**positive**	**positive**	positive
		4 weeks post-transplant	EDTA blood	**positive**	**positive**	**positive**	positive
		2.5 months post-transplant	EDTA blood	negative	negative	negative	negative†
		3.5 months post-transplant	EDTA blood	negative	negative	negative	negative†
		7 months post-transplant	EDTA blood	negative	negative	negative	negative
8	reactivation due to AIDS	first sample	EDTA blood	**positive**	**positive**	**positive**	positive
		sample 1 month later	EDTA blood	**positive**	**positive**	**positive**	positive†
		sample 1 month later	CSF	**positive**	**positive**	**positive**	positive†
9	reactivation due to AIDS	N/A	EDTA blood	**positive**	**positive**	**positive**	positive

All of the patients in Table 3 tested positive in serology for Chagas disease and no decrease in antibody titer was detected during the monitoring period.

BC = buffy coat; only the buffy coat fraction was PCR positive for these samples.

**†:** indicate specimens that were collected during drug treatment (benznidazole or nifurtimox). Drug treatment information is missing for patients #4 and #9.

**Table 4:** Detailed PCR-findings for recipients of organs from donors with suspected/confirmed chronic Chagas disease.

Patient ID	Patient description	time of specimen	Specimen type	results individual assays			Reported result
				kDNA TaqMan	TCZ TaqMan	18 S rRNA TaqMan	
10	kidney recipient	2 weeks post-transplant	EDTA blood	negative	negative	negative	negative
		3 weeks post-transplant	EDTA blood	**positive**	**positive**	negative	equivocal
		4 weeks post-transplant	EDTA blood	**positive**	**positive**	negative	equivocal
		5 weeks post-transplant	EDTA blood	**positive**	negative	negative	equivocal
		6 weeks post-transplant	EDTA blood	negative	negative	negative	negative
		7 weeks post-transplant	EDTA blood	negative	negative	negative	negative
		9 weeks post-transplant	EDTA blood	negative	negative	negative	negative
		11 weeks post-transplant	EDTA blood	negative	negative	negative	negative
11	kidney recipient	1 week post-transplant	EDTA blood	negative	negative	negative	negative
		3 weeks post-transplant	EDTA blood	**positive**	**positive**	negative	equivocal
		4 weeks post-transplant	EDTA blood	negative	negative	negative	negative
		5 weeks post-transplant	EDTA blood	negative	negative	negative	negative
		3 months post-transplant	EDTA blood	negative	negative	negative	negative
		6 months post-transplant	EDTA blood	negative	negative	negative	negative
12	Heart recipient	2 weeks post-transplant	EDTA blood	negative	negative	negative	negative
		3 weeks post-transplant	EDTA blood	negative	negative	negative	negative
		4 weeks post-transplant	EDTA blood	**BC positive**	**BC positive**	negative	positive
		5 weeks post-transplant	EDTA blood	**positive**	**positive**	negative	positive
		6 weeks post-transplant	EDTA blood	negative	negative	negative	negative†
		7 weeks post-transplant	EDTA blood	negative	negative	negative	negative†
		3 months post-transplant	EDTA blood	negative	negative	negative	negative
		5 months post-transplant	EDTA blood	negative	negative	negative	negative
		8 months post-transplant	EDTA blood	negative	negative	negative	negative

All of the patients in Table 4 were serologically negative at time of transplant and none sero-converted during the time they were monitored at CDC.

BC = buffy coat; only the buffy coat fraction was PCR positive for these samples.

**†:** indicate specimens that were collected during drug treatment (benznidazole or nifurtimox). Drug treatment information is missing for patients #4 and #9.

**Table 5:** Detailed PCR findings for patients with acute Chagas disease.

Patient ID	patient description	time of specimen	specimen type	results individual assays			Reported results
				kDNA TaqMan	TCZ TaqMan	18 S rRNA TaqMan	
13	acute infection after triatomine contact in endemic region^1^	sample 1	EDTA blood	**positive**	**positive**	**positive**	**positive**
		1 week later	blood clot	**positive**	**positive**	**positive**	**positive** †
		3 weeks later	EDTA blood	**positive**	**positive**	**positive**	**positive** †
		2 months later	EDTA blood	negative	negative	negative	negative†
		3 months later	EDTA blood	negative	negative	negative	negative
		4 months later	EDTA blood	**positive**	**positive**	negative	positive
		4.5 months later	EDTA blood	**positive**	negative	negative	equivocal
14	congenital transmission^2^	19 days old	EDTA blood	**positive**	**positive**	**positive**	**positive**
		2 months old	EDTA blood	negative	negative	negative	negative†

1 Patient was serologically positive for Chagas disease by the time she was tested at CDC.

2 Patient was serologically positive when 19 days old due to maternal antibodies. Another sample collected at 10 months of age tested negative in serology.

**†:** indicate specimens that were collected during drug treatment (benznidazole or nifurtimox).

### Detecting reactivation disease in immunosuppressed patients using real-time PCR

Specimens from 25 patients with chronic Chagas disease (as determined by positive serology) were received for evaluation of reactivation disease during the study period. Eighteen of the patients had received a heart transplant, five were HIV infected and two had undergone a bone marrow transplant. Sixteen patients had one or more PCR-positive samples, including sporadic PCR-positive results in seven patients who had received a heart transplant prior to 2008. Table 3 lists a selection of the samples analyzed from the remaining nine patients with at least one PCR-positive test result during this study. Seven patients were tested for reactivation following transplants; four of these were monitored on a regular basis by PCR. Two of the five HIV-positive patients were diagnosed with re-activated Chagas disease (patients 8 and 9); one of them had cerebral Chagas, confirmed by the presence of *T. cruzi* DNA in CSF.

### Using real-time PCR to detect T. cruzi transmission via organ transplants

Real-time PCR was used to test blood specimens from 14 previously non-infected transplant patients who received organs from a donor with suspected or confirmed chronic Chagas disease. It is recommended to closely monitor these patients with PCR or other sensitive technique in order to detect potential transmission as soon as possible. Three organ recipients had one or more PCR positive results (see Table 4). However, only patient 12, a heart recipient, was actually infected with *T. cruzi*. The PCR positive results for the other two patients (patients 10 and 11) were reported as equivocal and were most likely false positive results because of the following circumstances. Patient 10 received a kidney from a donor with borderline positive serology results with the Ortho *T. cruzi* ELISA test (Ortho-Clinical Diagnostics, Raritan, New Jersey). Since this could have been interpreted as indicative of infection in the donor, regular PCR testing was started on patient 10. However, subsequent serology testing of the donor associated with this case could not confirm the preliminary results; i.e., the *T. cruzi* RIPA was indeterminate and both the Wiener and the IIF test were negative on repeated serum samples. It was therefore concluded that the donor was not infected with *T. cruzi* and additional PCR follow up of patient 10 was unnecessary. However, before the final donor serology status had been determined, weak positive signals in the kDNA and TCZ TaqMan assays were verified in blood samples from patient 10. Unexpectedly, each subsequent specimen obtained from this patient showed a signal that was weaker than the signal obtained for the previous sample; i.e. the opposite of what was expected from an acute *T. cruzi* infection in an immunocompromised patient. At six weeks post-transplant patient 10 was no longer positive in any of the real-time PCR assays. Patient 11 received a kidney from a donor that was confirmed to be serologically positive for *T. cruzi*. The blood sample from patient 11 collected on the 3^rd^ week post-transplant tested weakly positive in the kDNA and TCZ TaqMan assays, with only the whole blood aliquot being positive and not the buffy coat fraction. The blood collected a week later was PCR negative in both whole blood and buffy coat. Neither patient 10 nor 11 had any clinical signs of *T. cruzi* infection. Their blood smears were constantly negative for parasites and they did not receive anti-trypanosomal drugs.

### Using real-time PCR to diagnose acute infections

We received 48 blood samples from 13 healthy patients who had been bitten or in close contact with triatomine bugs plus 9 laboratory workers that had been accidentally exposed to *T. cruzi* via needle stick accidents or animal bites during research activities. None of these were PCR positive. We tested 22 specimens from 20 children (aged newborn to 8 years) with sero-positive mothers for possible congenital transmission and detected *T. cruzi* DNA in the blood of a 19-days-old infant (patient 14 in Table 5). Twenty-eight specimens were received from 18 adult patients with symptoms of acute *T. cruzi* infection (fever and malaise after traveling to endemic region and/or having close contact with triatomine bug; three had a swollen eye that could be chagoma). Only one of these patients tested positive for *T. cruzi* by PCR and was treated for acute Chagas disease (patient 13 in Table 5). Follow-up specimens from this patient again tested positive in PCR after completed drug treatment but unfortunately the patient was lost to follow-up.

### Effect of buffy coat examination on the sensitivity of PCR-based detection

Thirty-five of the PCR-positive blood specimens (from 16 patients) had enough volume to allow for DNA extraction from both whole blood aliquots and buffy coat fraction. Of these, 26 specimens (from 10 patients) had PCR-detectable levels of *T. cruzi* DNA in both whole blood and buffy coat, with a relatively higher concentration in the buffy coat based on the quantitative output (the Cq value) from the real-time PCR assays. The remaining 9 specimens (from 6 patients) were positive only in the buffy coat fraction. Thus, 26% of the PCR-positive specimens would have been reported as being negative for *T. cruzi* if no buffy coat analysis had been performed. For three patients the analysis of buffy coat was crucial: Chagas disease reactivation in two patients was detected two weeks earlier by testing the buffy coat sample as compared to whole blood (patients 3 and 6 in Table 3) and the patient who acquired Chagas disease through transplantation (patient 12 in Table 3) was identified as positive one week earlier by testing buffy coat as compared to whole blood.

**Figure 1:**
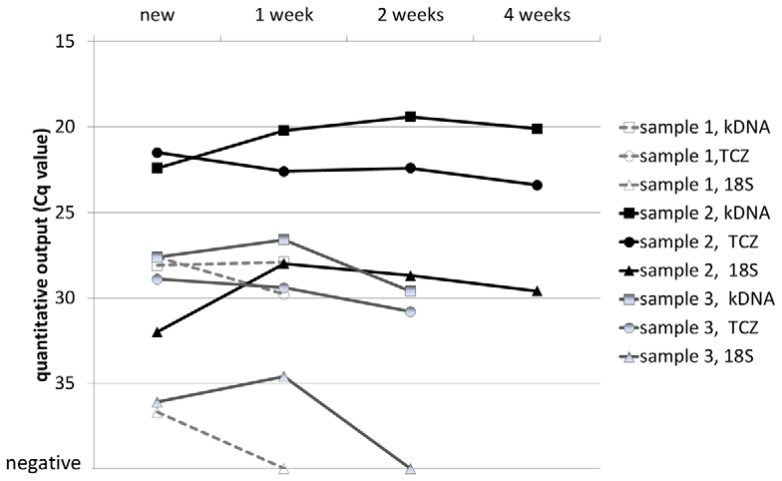
Effect of storage of EDTA blood specimens on real-time PCR results. The lower the Cq (quantitative cycle) value, the better recovery was obtained from the DNA extraction process.

Three of the PCR-positive blood samples had enough volume to allow for analysis of more than one buffy coat preparation. Aliquots of these three samples were stored at 4°C for up to four weeks and then processed as described. Figure 1 depicts the quantitative real-time PCR results obtained from these samples over time. The results suggested that storage of EDTA blood for a limited time had minor effect on the quality of *T. cruzi* DNA obtained from buffy coat preparations, at least for the kDNA and TCZ genetic regions. Although these are only preliminary data that need confirmation with a larger set of samples, it removes some of the uncertainty whether to accept EDTA-blood samples that for various reasons are delayed in transport to the diagnostic laboratory.

## Discussion

The laboratory diagnosis of Chagas disease relies mainly on serology, microscopic identification of trypomastigotes in blood or buffy coat, hemoculture and PCR [3]. Several PCR assays with variable diagnostic sensitivity and specificity have been developed and used as diagnostic tests [4], [7], [8], [9], [10], [11], [15], [16], [21]. A complicating factor for PCR assays is the high genetic variability of *T. cruzi* strains; there are currently six genotype groups or discrete typing units (DTU) described that differ significantly in genetic content and gene copy numbers [25], [26]. Since some DTUs are more common than others in various endemic regions, the same PCR assay can perform differently depending on the geographic origin of the specimen [16], [25], [27], [28], [29]. One way to circumvent these accuracy problems is to combine two or more PCR assays that target different genes.

The reference diagnostic laboratory at CDC employs a multi-target PCR testing algorithm consisting of three real-time PCR assays that are performed in parallel on all specimens. The three assays target different genomic regions in *T. cruzi* and have therefore variable sensitivity and specificity. The rationale for including all three assays in the testing algorithm is to ensure the highest accuracy possible by combining assays that complement each other. The kDNA TaqMan assay seems to be the most sensitive assay but it can amplify non-*T. cruzi* DNA, e.g. *T. rangeli*, and thus lead to false positives. The TCZ TaqMan assay has better specificity but as shown in this study can produce false positive PCR results as well. The kDNA and TCZ TaqMan assays are both much more sensitive than the 18 S rRNA TaqMan but the main advantage of including the 18 S rRNA assay in the testing algorithm is that it seems to be 100% specific. According to the CDC protocol, if a specimen tests positive in all three real-time PCR assays it will be reported as positive for *T. cruzi*, but any specimen that is only positive in the kDNA and/or TCZ TaqMan assays and negative in the 18 S rRNA TaqMan assay require additional confirmation by other tests or clinical data in order to be reported as positive for *T. cruzi*. If confirmatory data is absent or does not support a diagnosis of *T. cruzi* infection, the PCR results are reported as equivocal and a new specimen is requested to repeat the molecular analysis.

Diagnostic sensitivity can be enhanced by maximizing the amount of target DNA in the aliquot used for DNA extraction. For multi-copy PCR targets this can be obtained by mixing blood specimens with guanidine HCl-EDTA solution that lyses the parasites and releases their genetic content, thus making it possible to detect as little as one parasite in a large volume of blood [30], [31]. It has also been reported that sensitivity could be enhanced if blood clot was used as starting material [32]. An alternative method is to concentrate the parasites in the buffy coat fraction [33] prior to DNA extraction; this has been reported to increase the sensitivity compared to analysis of frozen EDTA-blood and guanidine HCl-EDTA treated blood [32], [34]. During this study, we compared the PCR results obtained from buffy coat concentration with results from fresh EDTA- blood and found that analysis of buffy coat allowed earlier detection of increasing levels of circulating parasite genome in three cases: two reactivation cases and one organ-transmitted acute infection. Thus, appropriate drug treatment for these patients could be initiated 1–2 weeks sooner.

Analyzing both the buffy coat fraction and a whole blood aliquot in parallel can also give helpful information to ensure test validity and to troubleshoot suspicious false positive PCR results. DNA extraction from the buffy coat fraction of a blood sample containing *T. cruzi* trypomastigotes should produce more *T. cruzi* DNA than the corresponding volume of whole blood. If that is not the case, there could be a problem with the quality of the blood specimen, the DNA extraction process or the PCR accuracy. One of the false positive PCR results obtained in this study was immediately flagged as suspicious because only the whole blood fraction was positive while buffy coat was negative. Nevertheless, more data must be accumulated during a longer period of time for a more robust assessment about the advantages of analyzing both whole blood and buffy coat.

In conclusion, we propose that in reference laboratories with the adequate infrastructure, the use of two or more real-time PCR tests with different performance characteristics combined with the analysis of buffy coat and whole blood can strengthen the use of PCR for accurate diagnosis of Chagas disease.

## Supporting Information

Checklist S1 **STARD checklist.**
(DOC)
